# Accelerating the Curing of Hybrid Poly(Hydroxy Urethane)-Epoxy Adhesives by the Thiol-Epoxy Chemistry

**DOI:** 10.1021/acsapm.2c01195

**Published:** 2022-11-16

**Authors:** Alvaro Gomez-Lopez, Bruno Grignard, Iñigo Calvo, Christophe Detrembleur, Haritz Sardon

**Affiliations:** † POLYMAT and Department of Polymers and Advanced Materials: Physics, Chemistry and Technology, Faculty of Chemistry, University of the Basque Country UPV/EHU, Paseo Manuel de Lardizabal 3, 20018Donostia-San Sebastián, Spain; ‡ Center for Education and Research on Macromolecules (CERM), CESAM Research Unit, University of Liège, allée du 6 août, Building B6A, Agora Square, 4000Liège, Belgium; § R&D Department, ORIBAY Group Automotive S.L., Portuetxe bidea 18, 20018Donostia-San Sebastián, Spain

**Keywords:** adhesives, poly(hydroxy urethane)s (PHUs), non-isocyanate polyurethanes (NIPUs), thiol-epoxy, click chemistry, hybrid materials, catalysts

## Abstract

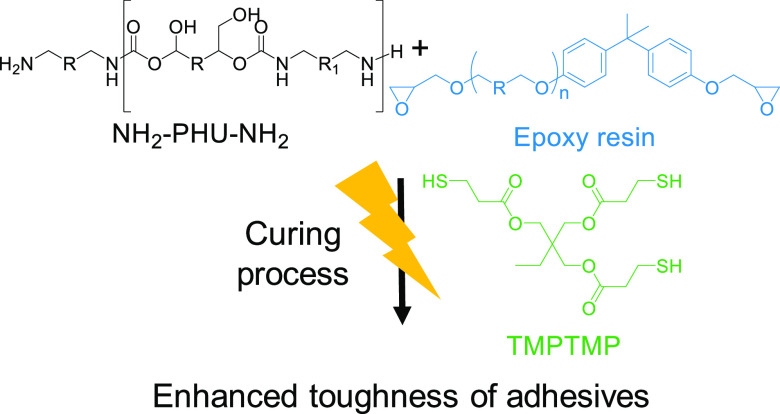

The polyaddition between dicyclic carbonates and diamines leading to poly(hydroxy urethane)s (PHUs) has emerged as the preferred method for the synthesis of green, non-isocyanate polyurethanes. However, when proposed for use as structural adhesives, the long times for completion of aminolysis of the 5-membered cyclic carbonates under ambient conditions force the use of complementary chemistries to accelerate the curing process. In this work, a system that combines an amino-terminated PHU (NH_2_–PHU–NH_2_), an epoxy resin, and a thiol compound was employed to develop high-shear strength PHU-epoxy hybrid adhesives able to cure at room temperature in short times. A NH_2_–PHU–NH_2_ prepolymer synthesized by using a sub-stoichiometric quantity of dicyclic carbonates was mixed with a bisphenol A-based epoxy resin for the preparation of the structural adhesive. While this adhesive showed good lap-shear strength and shear resistance under static load and temperature, the curing process was slow. In order to speed up the curing process, a thiol (trimethylolpropane tris(3-mercapto propionate)) was added and its impact on the curing process as well as on the adhesive properties was evaluated. The trifunctional thiol additive allowed for faster curing in the presence of the 1,1,3,3-tetramethylguanidine basic catalyst. Moreover, a combination of NH_2_–PHU–NH_2_ and the thiol as curing agents for the epoxy resin resulted in adhesives with superior toughness, without any deterioration of the ultimate lap-shear strength or shear resistance under load and temperature, making these adhesives suitable for high-demand applications in the automotive industry.

## Introduction

Polymer-based adhesives are widely employed in the automotive industry as they allow for a reduction in the weight of the final product and also increase the safety in terms of energy absorption when compared to spot welding, riveting, or clinching.^1,2^ Liquid structural adhesives are usually preferred for this purpose, although long curing times can become a major issue on assembly lines. Some technologies have decreased cycle times from hours to minutes, especially when (high) temperatures can be applied, making adhesives more cost-effective. It has to be noted that the automotive part manufacturers’ process is not adapted to the adhesive, but rather it is the adhesive that is adapted to the needs of the automotive production lines to get the best performance.

This automotive and transportation industry constitutes the largest consumer/end-user application of polyurethane (PU) adhesives, for which the market size is projected to grow to USD 9.1 billion by 2024 (at a 5.6% compound annual rate growth).^3^ Their excellent performance, flexibility, and ability to cure under ambient conditions in short times make PU-based adhesives an appealing option in this field. Nonetheless, the hazardous monomers, particularly isocyanates, which are demonstrated to provoke different illnesses^4−6^ as well as the chemicals related to their synthesis, such as phosgene,^7^ are at odds with current global sustainability trends. Thus, the hunt for harmless and greener alternatives to isocyanates represents one of the biggest challenges in PU industry nowadays.

Poly(hydroxy urethane)s (PHUs) prepared through the polyaddition reaction between dicyclic carbonates and diamines is one of the most popular routes to prepare green non-isocyanate PUs (NIPUs) due to the 100% atom economy of the process, the facile preparation of 5-membered cyclic carbonates through chemical [3 + 2] CO_2_ insertion into epoxy precursors, and the limited moisture sensitivity of these monomers.^8−10^ However, the slow aminolysis of 5-membered cyclic carbonates at room temperature forces researchers to employ high temperatures to prepare PHUs that compete with isocyanate-based PU adhesives.^11^ To overcome this limitation, hybrid systems that combine PHUs with other chemistries such as epoxy, sol–gel, or composites have been reported.^12^ The creation of hybrid systems through the combination with epoxy chemistry is particularly popular and has been shown to lead to PHUs with better adhesion performance^13−19^ and also to improve their properties for other applications, such as coatings.^20−24^

**Figure 1:**
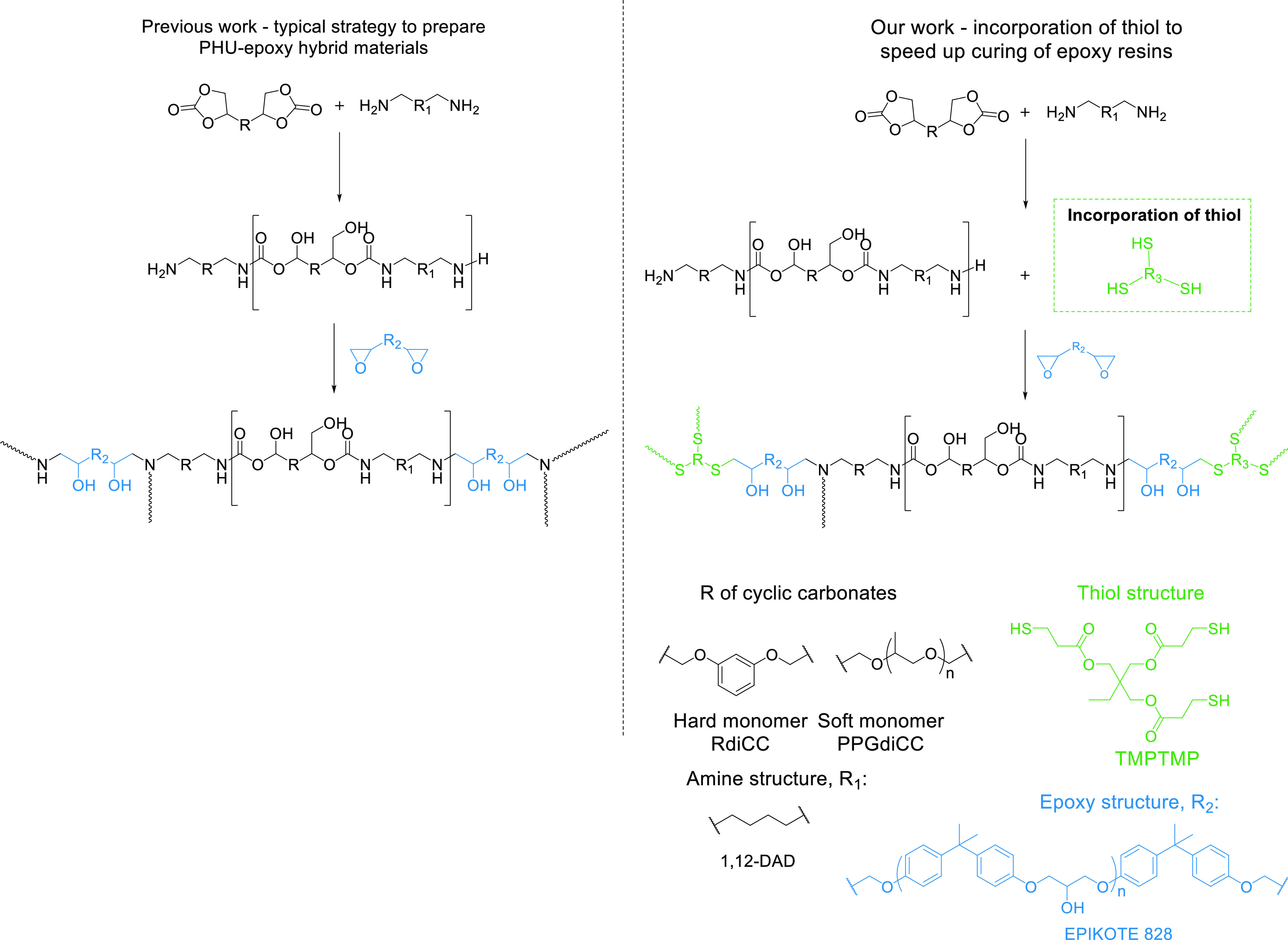
Conceptual strategy for the preparation of PHU-epoxy adhesives. Incorporation of thiol compounds highly speeded up the curing process.

There are two main strategies for the production of PHU-epoxy hybrids:^12,25,26^ (i) the one-step terpolymerization of epoxy and cyclic carbonates with amines and (ii) the chain extension of amino-terminated PHU (NH_2_–PHU–NH_2_) prepolymer with the epoxy resin. The amino-terminated prepolymer strategy is preferable as the reaction between amines and epoxides is much faster than the one between amines and cyclic carbonates (Figure 1). When performed in this way, the second step can be carried out at low or even room temperature in shorter times than one-step terpolymerization strategies.^27,28^

In our search for systems that could compete to isocyanate-based PUs and that displays enough reactivity at room temperature, we hypothesized that the curing process may be sped up by adding a new component to the formulation, a thiol compound to promote thiol-epoxy “click” chemistry (Figure 1). Thiol-epoxy polymerizations have industrial relevance in the area of adhesives, high-performance coatings, and composites.^29^ The reaction is rapid and selective and has 100% atom economy and takes place quantitatively in the bulk and under mild conditions.^29,30^ It is well established that the thiol-epoxy reaction catalyzed by strong bases such as imidazole and 1,1,3,3-tetramethylguanidine (TMG) takes place by a simple nucleophilic attack of the thiolate to the epoxy groups.^31,32^ The protonation of the alcoholate anions via either the base catalyst or the thiol gives rise to the final β-hydroxyl S-alkylated structure. A remarkable feature of thiol-epoxy condensation is that it can contribute to the acceleration of the initial hardening of the adhesive and decreasing the time required to reach handle strength. Meanwhile, NH_2_–PHU–NH_2_ will react more gradually and will contribute to the final mechanical properties of the adhesive.

In this work, PHU-thiol-epoxy hybrid adhesives are prepared and cured at room temperature using an epoxy resin with a combination of amino-terminated PHU and a thiol compound. First, the influence of the curing agent ratio, that is, PHU and thiol, on the curing step is explored through rheological measurements. Moreover, the impact of catalyst on this step is investigated by integrating a strong base, TMG, into the formulation. After assessing the curing parameters, the influence of curing agent ratios on ultimate adhesion performance was addressed by lap-shear and shear adhesion failure temperature (SAFT) measurements, while development of the lap-shear strength at different times was established through lap-shear tests. Additionally, physical characterization of the materials was performed by dynamic mechanical thermal analysis (DMTA), water uptake, and gel content measurements to evaluate the feasibility of the developed materials as structural adhesives.

## Experimental Section

### Reagents

Poly(propylene glycol) diglycidyl ether (*M*_n_ ∼ 640 g mol^–1^) (PPGDGE), resorcinol diglycidyl ether, tetrabutylammonium iodide (98%), trimethylolpropane tris(3-mercaptopropionate) (≥95.0%) (TMPTMP), 1,12-diaminododecane (98%) (1,12-DAD), and TMG (99%) were purchased from Sigma-Aldrich. 1,3-*bis*(2-hydroxyhexafluoroisopropyl)benzene (97%) was purchased from Fluorochem. Viscous epoxy resin based on bisphenol A (trade name: EPIKOTE Resin 828) and stainless steel AISI 316 were kindly supplied by Oribay Group. Deuterated dimethylsulfoxide (DMSO-*d*_6_) and methanol (MeOH-*d*_4_) were purchased from Sigma-Aldrich and Eurisotop, respectively. All reagents were used without further purification. The cyclic carbonates employed for this work were synthesized by CO_2_ coupling with commercial precursors as reported elsewhere.^33^

### Titration of the Cyclic Carbonate by ^1^H NMR

Titration of cyclic carbonates was performed as described elsewhere.^34^ Carbonate equivalent weights (CEWs) of PPGdiCC and RdiCC were calculated according to eq 1, where *m*_C5_ is the mass of cyclic carbonate introduced into the nuclear magnetic resonance (NMR) tube; *n*_function of carbonate_ is the molar amount of function carbonate in cyclic carbonate; *I*_a_, *I*_b_, and *I*_c_ are integrations of characteristics peaks *a*, *b*, and *c* of carbonate, respectively; *n*_toluene_ is the molar amount of toluene introduced in standard solution; and *I*_CH3_ is the integration of peak CH_3_ of toluene. The CEW values for each cyclic carbonate were obtained in triplicate determinations and are presented in Table S1
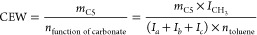
1

### Typical Procedure for Amino-Terminated PHU Oligomer Preparation

PPGdiCC (5.3032 g, 15.91 × 10^–3^ equiv), RdiCC (2.0212 g, 10.60 × 10^–3^ equiv), and 1,12-DAD (5.3119 g, 53.02 × 10^–3^ equiv) were added to a 100 mL round-bottom flask equipped with a half-moon Teflon helix stirrer and put into an oil bath at 80 °C. Mechanical stirring at 100 rpm was kept for 5 h. Then, the stirring was stopped, the reaction was cooled down, and amino-terminated PHU oligomers were obtained and used for the further preparation of H-NIPU materials without purification.

Equation 2 was employed for the calculation of the amine mass. The molar ratio between PPGdiCC/RdiCC was fixed to 1.5 (based on previous research and experience),^34−36^ and the molar ratio of amine with respect to cyclic carbonates was fixed to 2 (*r* = 2). The number of active hydrogens of amine was fixed at 1 for one amine function reacting with one cyclic carbonate. The active hydrogen equivalent weight (AHEW) was calculated from eq 4
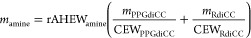
2

### Titration of the NH_2_–PHU–NH_2_ by ^1^H NMR

The amine equivalent weight (AEW) is the amount of resin (in grams) containing 1 g equivalent of amine functions. It was determined following the procedure used for the determination of the CEW. Toluene standard solution was prepared in MeOH-*d*_4_. The characteristic signal at 2.65 ppm of the methylene group next to the free amine was chosen. The AEW is calculated using eq 3, where  and  correspond to the integrals of the CH_3_ of toluene and of the two methylene protons of NH_2_–PHU–NH_2_, respectively. Measurements were replicated three times
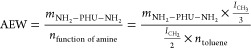
3

The amine hydrogen equivalent weight (AHEW) for primary amines was calculated from eq 4, taking into account the reactive hydrogens for each type of reaction. For example, in the case of the polyaddition with cyclic carbonates, amines present one active hydrogen, while for the preparation of hybrid materials through reaction with epoxies, amine has two active hydrogens
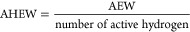
4

### Typical Procedure for the Synthesis of Hybrid Materials

The synthesis of a 70/30 composition is described as a typical procedure for the preparation of hybrid materials. Amino-terminated PHU oligomer (1.0706 g, 4.12 × 10^–3^ equiv), TMPTMP (0.2468 g, 1.76 × 10^–3^ equiv), and EPIKOTE 828 (1.1000 g, 5.88 × 10^–3^ equiv) were weighed in a 26 mL vial. The reactants were stirred for 2 min at 50 °C, followed by 1 min at room temperature to obtain a homogeneous mixture. Then, the viscous oligomeric solution was transferred into a plastic 6 mL syringe for applying onto the substrates for the preparation of lap-shear (∼80 mg) or SAFT (∼200 mg) joints.

For the preparation of 0/100 composition, EPIKOTE 828 (1.5535, 8.32 × 10^–3^ equiv) was first mixed with TMG (9.12 μL, 7.27 × 10^–5^ equiv) and then, TMPTMP (1.1631 g, 8.32 × 10^–3^ equiv) was added. Mixing was done as previously explained. The homogeneous mixture was applied onto substrates and second substrates were placed after 20 min, allowing the adhesive to get enough viscosity to avoid spreading out from the bonding line. The addition of TMG was necessary as epoxy and thiol compounds do not react without the presence of a catalyst.

### Characterization and Methods

#### Nuclear Magnetic Resonance

^1^H spectra were recorded on a Bruker Advance DPX 300 spectrometer at 25 °C. DMSO-*d*_6_ and MeOH-*d*_4_ were used as solvents.

#### Fourier Transform Infrared Spectroscopy

Fourier transform infrared (FT-IR) spectra were obtained using an FT-IR spectrophotometer (Nicolet is20 FT-IR, Thermo Scientific Inc., USA) equipped with attenuated total reflection (ATR) with a diamond crystal. Spectra were recorded between 4000 and 525 cm^–1^ with a spectrum resolution of 4 cm^–1^ at room temperature. All spectra were averaged over 16 scans.

#### Rheology Measurements

Time sweep experiments were performed in a stress-controlled Anton Paar Physica MCR101 rheometer at room temperature at a frequency of 1 Hz and a strain of 1% in order to determine the crossover between loss modulus (*G*″) and storage modulus (*G*′) (gel time). The experiments were carried out using 15 mm disposable parallel plate geometry.

#### Dynamic Mechanical Thermal Analysis

DMTA experiments were performed using a rectangular sample of the crosslinked materials (2 × 3.5 × 1 mm) using a Triton 2000 DMA from Triton Technology in single cantilever bending deformation mode. Tests were performed at 1 Hz at a heating rate of 4 °C min^–1^ from −35 to 110 °C. Crosslinking density () was calculated from eq 5 on the basis elasticity theory^37^
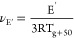
5where *E*′ is the storage modulus at *T*_g+50_, *R* is the gas constant, and *T*_g+50_ is the temperature, in K, 50 K above *T*_g_ (determined at the maximum of the tan δ curve).^38^

#### Swelling Index

The swelling index was measured after Soxhlet extractions in refluxing THF for 24 h, wiping samples to remove residual THF before weighing. Values were calculated with
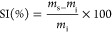
6where *m*_s_ is the weight of the swollen sample and *m*_i_ is the initial weight of the sample.

#### Gel Content

The gel content was measured by Soxhlet extraction in refluxing THF for 24 h. Afterward, samples were dried in oven at 70 °C for 24 h. Values were calculated with
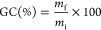
7where *m*_f_ is the final weight of the dried sample and *m*_i_ is the initial weight of the sample.

#### Equilibrium Water Content

Three samples (∼30 mg) of each formulation were immersed separately into 10 mL of deionized water for 96 h. The equilibrium water content (EWC) was calculated by
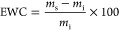
8where *m*_s_ is the weight of the swollen sample and *m*_i_ is the initial weight of the sample.

#### Lap-Shear and SAFT Tests

Lap-shear and SAFT tests were performed following the procedure as reported elsewhere.^34^ For lap-shear test, a parallel force to the adhesive bond with a displacement rate of 1 mm min^–1^ was applied. The adhesive joints were allowed to cure at ambient temperature for 24 h prior testing. Addressing the evolution of the lap-shear strength over time, samples were allowed to cure the corresponding time (1, 2, 3, 5, 7, and 24 h).

## Results and Discussion

**Figure 2:**
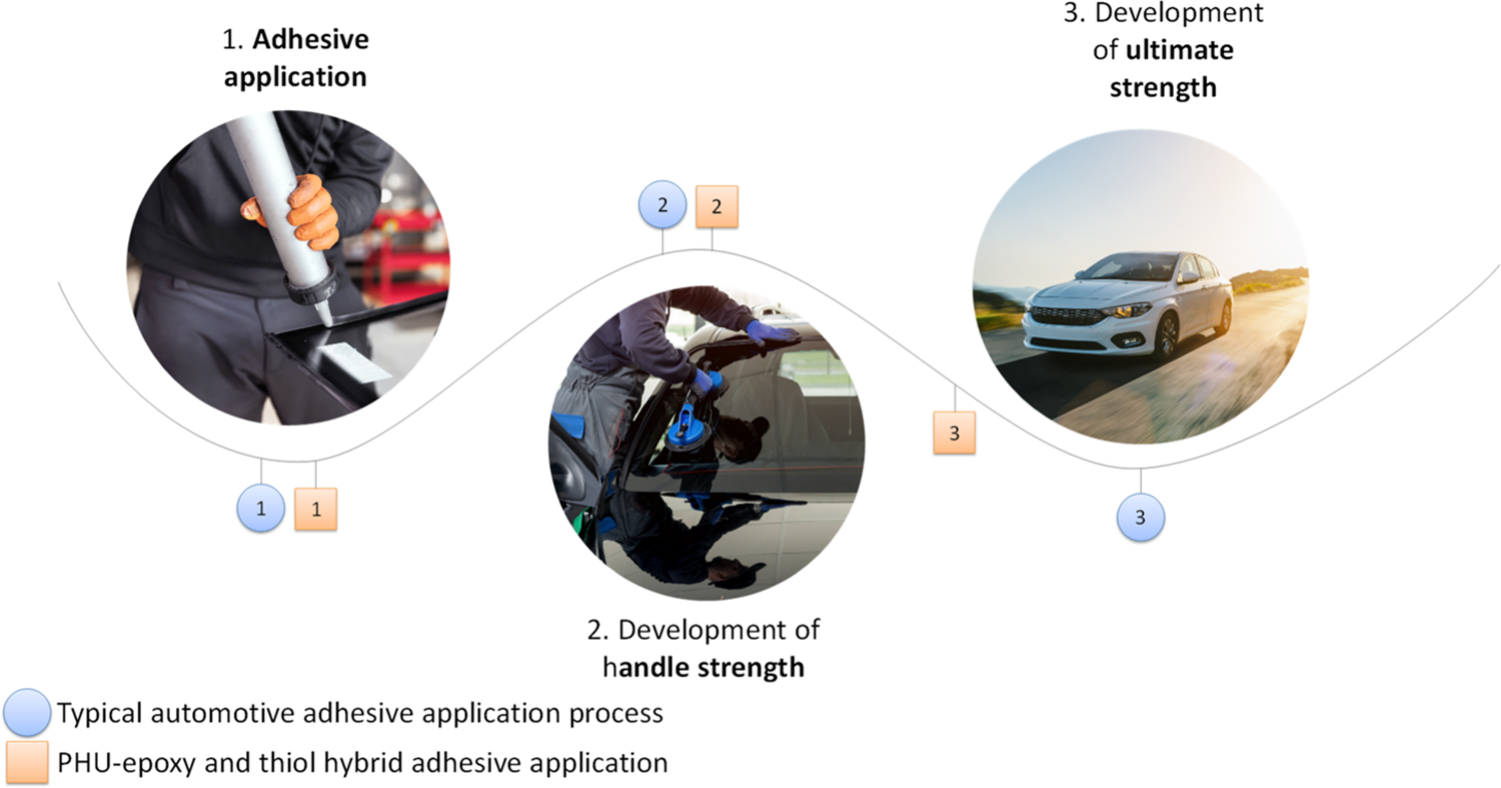
Differences between the typical automotive adhesive application process (blue) and its acceleration by the addition of thiol into the formulation (orange). Reproduced with permission from herraez/stock.adobe.com, romaset/stock.adobe.com, and AA + W/stock.adobe.com.

As mentioned in the introduction, in the automotive sector, the adhesive must be designed by considering the requirements of the automotive production lines. In particular, liquid structural adhesives that are used in production plants must harden as fast as possible to achieve enough strength in order to hold the parts of the ensemble together (handle strength). Afterward, the adhesive will remain there until it reaches the ultimate strength of the adhesive joint related to the final application properties (Figure 2).

When designing NIPU-type materials for this type of application, it is clear that the reaction between 5-membered cyclic carbonates and diamines is not fast enough to meet the industrial specifications and that the maximal strength values are not comparable to the ones of isocyanate-based PU adhesives. The enhancement in the ultimate strength values has been solved by the use of hybrid chemistries such as epoxy chemistry. However, while several different chemistries have been used for curing NIPUs at room temperature,^12,25^ in all cases, the process is still relatively slow and long times are required to obtain a minimum handle strength. We envisioned that the curing process may be sped up by adding a new component to the formulation. Therefore, in the first part of the article, we have prepared some NH_2_–PHU–NH_2_ oligomers that are further combined with an epoxy resin and a multifunctional thiol. The effect of these components on the curing process as well as on the adhesion properties is evaluated.

### Synthesis of Amino-Terminated PHU Oligomers

Based on our previous work,^34−36^ a mixture of balanced soft to hard segment monomers, that is, PPGdiCC/RdiCC in 60:40 molar ratio, was reacted with 2 equiv of 1,12-DAD at 80 °C for 5 h under continuous mechanical stirring for the preparation of an amino-terminated PHU. FT-IR–ATR was used for tracking this reaction by following the disappearance of the carbonyl stretching vibration band of the cyclic carbonates. After 5 h, these bands (1790 and 1782 cm^–1^ of PPGdiCC and RdiCC, respectively) had totally disappeared and a new band appeared at 1701 cm^–1^, corresponding to the carbonyl of the urethane group, was clearly observed (Figure S1b). Additionally, vibration bands at 3304 (O–H, N–H), 1593 (C=C, ar), 1536 (δ N–H), 1492 (C=C, ar), 1463 (C–O), 1254 (C–O–C, as st), and 1101 cm^–1^ (C–O–C, sy st) overlapped with C–O stretching vibration bands of primary and secondary alcohols and at 772 and 721 cm^–1^ (C–H out-of-plane bending vibrations of the aromatic rings, meta substitution) confirmed the structure of the prepolymer. ^1^H NMR spectroscopy of NH_2_–PHU–NH_2_ (Figure S1c) showed the splitting of the signal at 2.65 ppm (NH_2_–CH_2_−) and the appearance of a new signal at 3.11 ppm (CH_2_–NH–C(O)–O), confirming the functionalization of the NH_2_–PHU–NH_2_. The AEW was determined by ^1^H NMR titration (Figure S2) in a standard solution of toluene in CD_3_OD as described in the experimental section. NH_2_–PHU–NH_2_ was characterized with an AEW of 520.4 ± 5.5 g equiv^–1^. Taking into account that amines possess two active hydrogens when reacting with epoxy resins, an AHEW of 265.2 ± 2.2 g equiv^–1^ was calculated from eq 4.

### Preparation of Hybrid PHU-Epoxy Adhesives

To synthesize PHU hybrid materials, EPIKOTE 828 resin (epoxy resin) was reacted with different equiv ratios of curing agents, that is, NH_2_–PHU–NH_2_ and TMPTMP. Thus, the sum of active hydrogen equivalents of NH_2_–PHU–NH_2_ and thiol equivalents of TMPTMP matched the equivalent number of epoxy group for all compositions. Five different formulations were prepared varying the NH_2_––HU-NH_2_/TMPTMP ratio (100/0, 70/30, 50/50, 30/70, and 0/100). Due to viscosity issues, in all cases, the reagents were mixed at 50 °C for 2 min, followed by 1 min at room temperature, to give a homogeneous viscous mixture.

**Figure 3:**
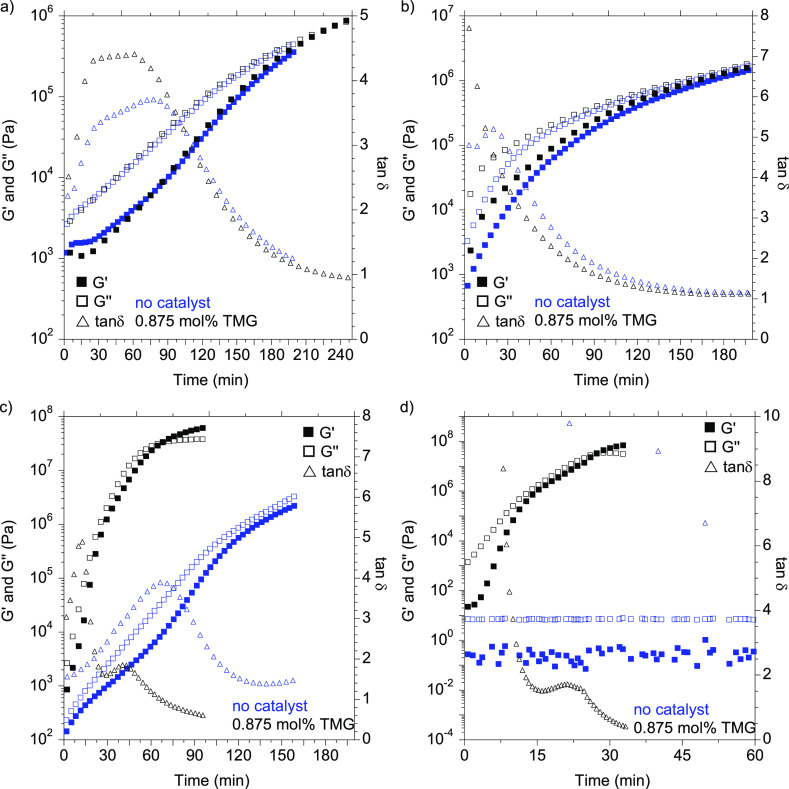
Evolution of *G*′, *G*″, and tan δ values over time obtained from rheological measurements carried out at 25 °C without the catalyst (blue symbols) and in the presence of 0.875 mol % of TMG (black symbols) of (a) 100/0, (b) 70/30, (c) 30/70, and (d) 0/100 NH_2_–PHU–NH_2_/TMPTMP equivalent ratio compositions mixed with 1 equivalent of EPIKOTE 828.

The ability of the materials to cure at room temperature was checked by rheology by determination of the gel time (*G*′ = *G*″) of the various reactive formulations. Catalyst-free compositions were characterized with gel times longer than 2 h when NH_2_–PHU–NH_2_ was employed, independent of the ratio of curing agents (Figure 3, blue spots). Despite the lower initial viscosity of the NH_2_–PHU–NH_2_/TMPTMP 30/70 composition, the rapid moduli increased attested for a faster curing. This can be attributed to the higher reactivity and functionality of the thiol. Indeed, the amine has to react twice with the epoxy to create a thermoset material and the secondary amine that is formed possesses a lower reactivity, resulting in slower formation of the crosslinked network. The 0/100 equiv ratio composition was not able to cure without adding a catalyst. The moduli as well as the stretching vibration bands of the S–H (2569 cm^–1^) and C–O–C of the epoxy (914 cm^–1^) remained unaffected after 2 h (Figure 3d, blue spots), and little change was observed on the timescale of 1 week (Figure S3). This result suggests that NH_2_–PHU–NH_2_ acts as a catalyst for the thiol-epoxy reaction. Ending of the reaction after 24 h was confirmed by FT-IR–ATR through the disappearance of the stretching vibration band of the thiol at 2569 cm^–1^ (Figure S4a) and the decreased intensity of the asymmetric C–O–C stretching vibration band of the epoxy at 914 cm^–1^ (Figure S4a). A shoulder in the zone of the C–O stretching vibration of the carbonyl group was observed for compositions in which NH_2_–PHU–NH_2_ and TMPTMP were employed due to the carbamate and the ester group, respectively.

**Figure 4:**
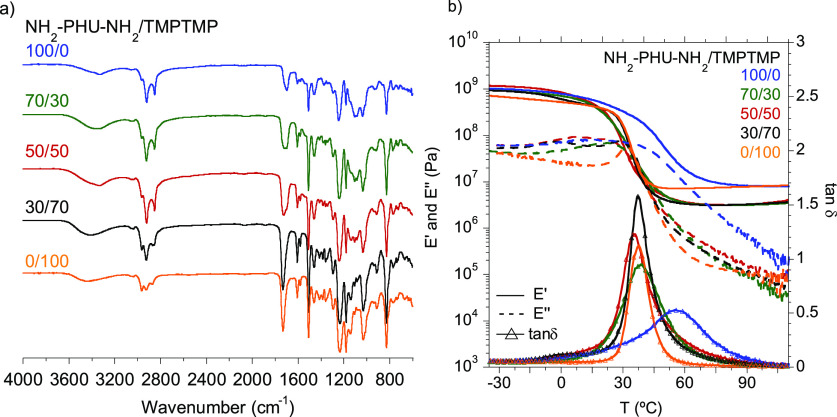
(a) FT-IR–ATR spectra of the different compositions of hybrid PHU-epoxy adhesives; (b) *E*′, *E*″, and tan δ values for the compositions of the hybrid PHUs.

The residual signal of the epoxy ring vibration band (Figure S4b) is due to vitrification of the system, which causes a dramatic lowering in the mobility of the polymer chains, and therefore, conversion of the system is limited.^39^ Nonetheless, the formation of a three-dimensional network was confirmed by DMTA experiments, in which *G*′ was over *G*″ at high temperatures (Figure 4b), and high crosslinking degrees were achieved (Table 1).

**Table 1:** Physical and Thermal Characterization of Hybrid PHU-Epoxy Adhesives

entry	composition (NH_2_–PHU–NH_2_/TMPTMP equiv ratio)	GC^a^ (%)	SI^b^ (%)	*T*_g_^c^ (°C)	*E*_Tg+50°C_^′^^d^ (MPa)	ν_*E*′_^e^(10^2^mol m^–3^)	(Pa s)^f^	*t*_gel_ (min)^f^
1	100/0	98.2 ± 0.0	83.9 ± 3.9	54.1 ± 1.5	8.3 ± 0.4	8.8 ± 0.4	406/386	>2 h^g^
2	70/30	99.0 ± 0.6	94.0 ± 3.0	38.9 ± 0.1	3.6 ± 0.6	4.0 ± 0.7	401/1210	>2 h^g^
3	50/50	86.0 ± 0.3	179.3 ± 10.2	34.4 ± 1.8	2.3 ± 1.2	2.6 ± 1.3	nd/nd	nd/nd
4	30/70	88.4 ± 0.8	122.0 ± 1.6	37.2 ± 0.2	2.9 ± 0.5	3.2 ± 0.5	35.5/264	>2 h/67
5	0/100	>99%	80.6 ± 3.4	36.7 ± 0.8	8.1 ± 2.1	9.0 ± 2.4	1.2/182	h/27

a Gel content.

b Swelling index after Soxhlet extraction in refluxing THF for 24 h.

c Glass-transition temperature from DMTA measurements.

d Storage modulus in the rubbery region of the material (*T*_g_ + 50 °C).

e Crosslinking density calculated from the rubbery plateau using eq 5.

f Values are referred to samples without/with the catalyst, respectively.

g In both cases, without and with the catalyst.

h No gel time was observed.

### Accelerating the Preparation of Hybrid PHU-Epoxy Adhesives

Some reports have shown that the epoxy thiol reaction can be sped up substantially by adding strong bases such as guanidines or amidines.^29^ Capitalizing on these features, we selected and incorporated a small amount of TMG (0.875 mol % with respect to equiv of epoxy group) as a catalyst within the reactive formulations. The curing process was found to be highly catalyst- and formulation-dependent. The thermoset material based on only thiol-epoxy chemistry was cured in less than 30 min, while in the absence of the catalyst, no curing was observed (Figure 3d, curve black). Besides, the addition of the TMG catalyst to a NH_2_–PHU–NH_2_/epoxy formulation did not accelerate the curing process, as highlighted in Figure 3a, showing similar *G*′ and *G*″ evolution over time with or without TMG. For the ternary compositions NH_2_–PHU–NH_2_/epoxy/TMPTMP, an increment was observed, which was more pronounced as the amount of thiol was increased. The composition containing 70 mol % of TMPTMP exhibited a huge decrease in gel time (76 min) when the catalyst was employed (Figure 3c), confirming that TMG catalyzed only the thiol-epoxy reaction.

### Physicochemical Characterization of the Hybrid PHUs

As mentioned in the introduction, liquid structural adhesives are characterized by two stages after application. The first is achieved when the adhesive possesses enough strength to hold together the parts to be adhered, whereas the second stage is reached when the adhesive has developed the final adhesive properties. Before measuring the adhesion properties, the physicochemical characterization of the adhesive was performed through DMTA and Soxhlet extraction (Table 1). The gel content measured by Soxhlet extraction was close to 90% or above in all cases, demonstrating the high degree of crosslinking. Moreover, the glass-transition temperatures were calculated from the temperature at the maximum value of tan δ. In all cases, similar *T*_g_ values were observed. However, when only NH_2_–PHU–NH_2_ was used as a curing agent in the formulation, a broader *T*_g_ was observed, indicating an increased level of heterogeneity in the adhesive, which may be due to the immiscibility between the polar NH_2_–PHU–NH_2_ and the non-polar epoxy resin.

Crosslinking densities were calculated at a temperature of 50 K above the *T*_g_ where the polymer is in the rubbery plateau.^37,38^ Interestingly, a minimum of ν_E′_ for 50/50 equiv ratio composition was observed. Our hypothesis is that two different zones are observed below and above 50% of PHU. At low PHU concentrations, substitution of short TMPTMP chains by longer NH_2_–PHU–NH_2_ oligomers resulted in a decrease in crosslinking density as a result of the increased distance between covalent crosslinking nodes. Taking into account this explanation, the NH_2_–PHU–NH_2_/TMPTMP 50/50 composition would be expected to present a higher crosslinking density than the analogous 70/30 and 100/0 compositions. Nonetheless, the opposite was observed and the 100/0 equiv ratio formulation presented more than a 2-fold increase in crosslink density. Our thought is that physical inter- and/or intramolecular hydrogen bonding between NH_2_–PHU–NH_2_ chains created strong interactions that enhanced the effective crosslinking density and decreased swelling of the polymer. The swelling index showed the opposite behavior to the crosslinking density; thus, the lowest crosslinking density correlated with a maximum in swelling index since penetration of the solvent into the network occurs more easily.

### Ultimate Adhesive Properties

**Figure 5:**
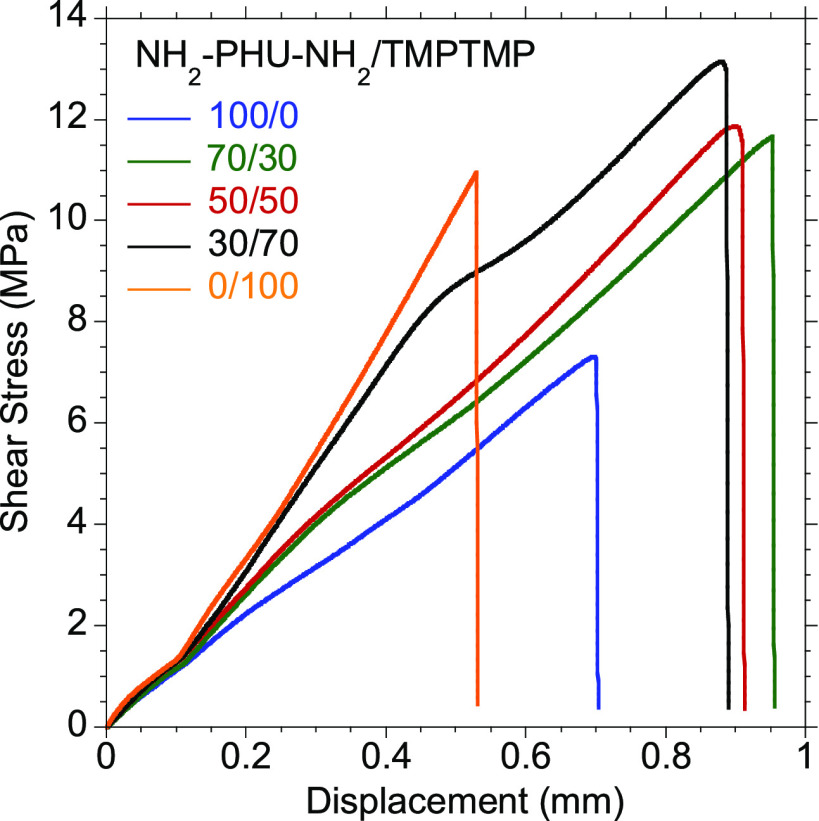
Representative shear stress–displacement curves for 70/30 (green), 50/50 (red), and 30/70 (black) NH_2_–PHU–NH_2_/TMPTMP equiv ratios after 24 h of curing at room temperature; for the compositions in which NH_2_–PHU–NH_2_ and TMPTMP were combined to cure the epoxy resin, similar values of ultimate lap-shear strength were achieved as for the TMPTMP-cured composition (0/100). This indicates that the addition of TMPTMP to the formulations enhanced the adhesive performance, even at lower equivalent amounts. On the other hand, a combination of curing agents exhibited a synergetic effect to increase the elongation at break of the adhesives. This indicates an enhancement of the toughness, without losing maximum force resistance, making the adhesive joint less brittle. It is worth noting that maximizing toughness is important in structural adhesives as it makes the adhesive joint more resistant to vibrations and shock impacts. The increase of the elongation at break can be related to the lower crosslinking densities (Table 1) of the formulations with combinations of curing agents (70/30, 50/50, and 30/70 NH_2_–PHU–NH_2_/TMPTMP), which makes the adhesive more elastic. Cohesive failures were observed for 50/50 and 30/70 compositions (Figure S5c,d respectively), meaning that the forces at the interface of the joint elements were greater than the intrinsic cohesive forces of the polymer. This failure is more preferable since adhesive joint breakage is easier to predict. The rest of the formulations presented adhesive failure (Figure S5a,b,e), showing greater cohesiveness than affinity to the substrate.

The ultimate adhesive properties of the hybrid PHUs were addressed by a lap-shear test (Figure 5). The NH_2_–PHU–NH_2_-epoxy mixture (Figure 5, blue curve) was characterized with a lap-shear strength of 7.6 ± 0.9 MPa, while for the thiol-epoxy mixture (Figure 5, orange curve), this value was increased up to 11.4 ± 1.1 MPa. In both cases, the adhesive remained on just one of the substrates after bond breaking, meaning a greater cohesiveness of the polymer chains than adhesion forces with the adherend (Figure S5a and e, respectively).

Besides shear strength under dynamic deformations (lap-shear tests), the evaluation of structural adhesives under the effect of static load and temperature is also important. These measurements give information about resistance of the material to creep and give an idea of viable service temperatures of the adhesives. To get this information, SAFT measurements were carried out (Table 2). In all cases, adhesives were able to withstand 217 °C more than 7 h, thus showing excellent resistance to creep at high temperatures. The epoxy resin is a key contributor to the good shear resistance of these formulations as can be seen from comparison of these results to our previous work with PHU adhesives.^35^

**Table 2:** Lap-Shear Strength and SAFT Values of the Compositions Cured at Room Temperature for 24 h

entry	composition (NH_2_–PHU–NH_2_/TMPTMP equiv ratio)	lap-shear strength (MPa)	SAFT^a^ (°C)
1	100/0	7.6 ± 0.9	217 ± 0^b^
2	70/30	11.6 ± 0.8	217 ± 0^b^
3	50/50	11.8 ± 1.0	217 ± 0^b^
4	30/70	12.8 ± 1.0	217 ± 0^b^
5	0/100	11.4 ± 1.1	217 ± 0^b^

a SAFTs at 1 kg/625 mm^2^.

b Maintained the adhesive properties at this temperature for more than 7 h.

### Evolution of Lap-Shear Strengths over Time

**Figure 6:**
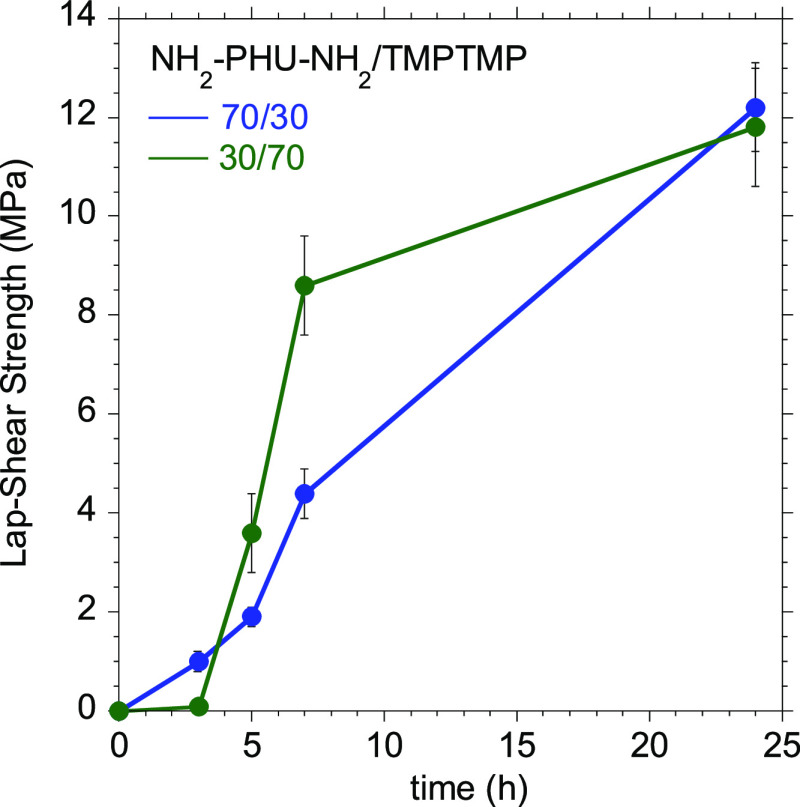
Evolution over time of the lap-shear strength values for the 70/30 and 30/70 compositions. Error bars represent standard deviation for each sample set.

In order to develop an industrially relevant adhesive, in addition to the ultimate adhesive properties, the evolution of the adhesive properties over time must be taken into account. The rapid development of a minimum lap-shear strength (1.2 MPa^40^ may be taken as a reference value for handling strength) is mandatory to be competitive in the industrial manufacturing process since this is the principal method for improving production speed and reducing costs. Therefore, lap-shear measurements were carried out at different times to evaluate the development of lap-shear strength for the 70/30 and 30/70 NH_2_–PHU–NH_2_/TMPTMP equivalent ratio compositions (Figure 6). These formulations were selected based on rheological results, in which they showed faster curing times without the catalyst than formulations just based on NH_2_–PHU–NH_2_ or TMPTMP as curing agents. The 70/30 composition showed a smoother evolution of the lap-shear strength (Figure 6, blue curve), developing handling strength (1 MPa of adhesion) after 3 h. On the other hand, a sharper evolution of the lap-shear strength was observed for 30/70 composition (Figure 6, green curve). This composition required a greater time to reach the minimum lap-shear strength for handling, but afterward, it surpassed the values of the 70/30 equivalent ratio formulation. After 7 h of curing, it reached up to 72.9% of the ultimate lap-shear strength.

## Conclusions

In this work, PHU-epoxy hybrid adhesives able to cure at room temperature were prepared by mixing an amino-terminated PHU (based on 60/40 molar ratio mixture of PPGdiCC and RdiCC reacted with excess of 1,12-DAD) with a viscous epoxy resin, EPIKOTE 828, and a trifunctional thiol (TMPTMP). NH_2_–PHU–NH_2_-epoxy hybrids cured at room temperature showed a lap-shear strength of 7.6 ± 0.9 MPa, which was enhanced up to 12.8 ± 1.0 MPa with the integration of a trifunctional thiol, TMPTMP. More significantly, the toughness of the adhesives was improved through the combination of both curing agents. Higher values of elongation at break were related to lower crosslinking densities and therefore greater elasticity of the adhesives. Thus, adhesives with enhanced resistance to vibrations and shock impacts were developed without losing adhesion performance. The adhesives also exhibited excellent resistance to shear under static load and high temperatures, tolerating 1 kg static shear load at 217 °C for more than 7 h. Lap-shear evolution over time showed that the 70/30 NH_2_–PHU–NH_2_/TMPTMP composition was able to develop the required handling strength within only 3 h at room temperature. With a view to speed up the reaction times, a strong base (TMG) was added to the composition to boost the rate of the thiol-epoxy “click” reaction. Despite rheological measurements demonstrating faster curing when just 0.875 mol % of TMG was added to the 30/70 composition, resulting in a gelation in 76 min, the adhesive could not be applied due to the low pot life time. Strategies to add TMG at the time of application are under development to accelerate the curing times without altering the pot life of the adhesive.

## Supporting Information Available

CEW, AHEW, EEW, and TEW values; structure; FT-IR–ATR and ^1^H NMR spectra of NH_2_–PHU–NH_2_; titration ^1^H NMR spectra; stability FT-IR–ATR spectrum; FT-IR–ATR spectra of the representative example of the evolution of the curing process; and nature of adhesive failure of adhesive joints (PDF)

## Abbreviations

ar: aromatic group
as st: asymmetric stretching vibration
sy st: symmetric stretching vibration
