# Supplementary material for: Accelerating the Curing of Hybrid Poly(Hydroxy Urethane)-Epoxy Adhesives by the Thiol-Epoxy Chemistry
Source: ACS Appl Polym Mater. 2022 Nov 16;4(12):8786–94. doi: 10.1021/acsapm.2c01195 (PMC9745729; doi:10.1021/acsapm.2c01195)
Supplement: Supplementary file 1 — ap2c01195_si_001.pdf [file ap2c01195_si_001.pdf]

Supporting Information - Accelarating the Curing of Hybrid Poly(Hydroxy Urethane)-Epoxy Adhesives by the Thiol-Epoxy Chemistry

Alvaro Gomez-Lopez[ʒ,ϒ], Bruno Grignard[ǂ], Iñigo Calvo[¥], Christophe Detrembleur[ǂ], Haritz Sardon[ʒ,*]

[ʒ]POLYMAT and Polymer Science and Technology Department, Faculty of Chemistry, University of the Basque Country UPV/EHU, Paseo Manuel de Lardizabal 3, 20018, Donostia-San Sebastián, Spain. haritz.sardon@ehu.eus

[ǂ]Center for Education and Research on Macromolecules (CERM), CESAM Research Unit, University of Liège, allée du 6 août, Building B6A, Agora Square, 4000 Liège, Belgium.

[¥]ORIBAY Group Automotive S.L. R&D Department, Portuetxe bidea 18, 20018, Donostia-San Sebastián, Spain.

Present address

[ϒ]ORIBAY Group Automotive S.L. R&D Department, Portuetxe bidea 18, 20018, Donostia-San Sebastián, Spain

Table S1. Carbonate equivalent weight (CEW), active hydrogen equivalent weight (AHEW), epoxy equivalent weight (EEW) and thiol equivalent weight values.

Reactant	CEW (g/eq.)	AHEW (g/eq)	EEW (g/eq)	TEW (g/eq)
PPGdiCC	333 ± 4	-	-	-
RdiCC	190 ± 5	-	-	-
1,12-DAD	-	100.18	-	-
NH_2-PHU-NH_2	-	260.2 ± 2.8	-	-
TMPTMP	-	-	-	132.85
EPIKOTE[TM] 828	-	-	184-190[a]	-

[a] Value provide by Hexion. The media between values was employed for calculations.

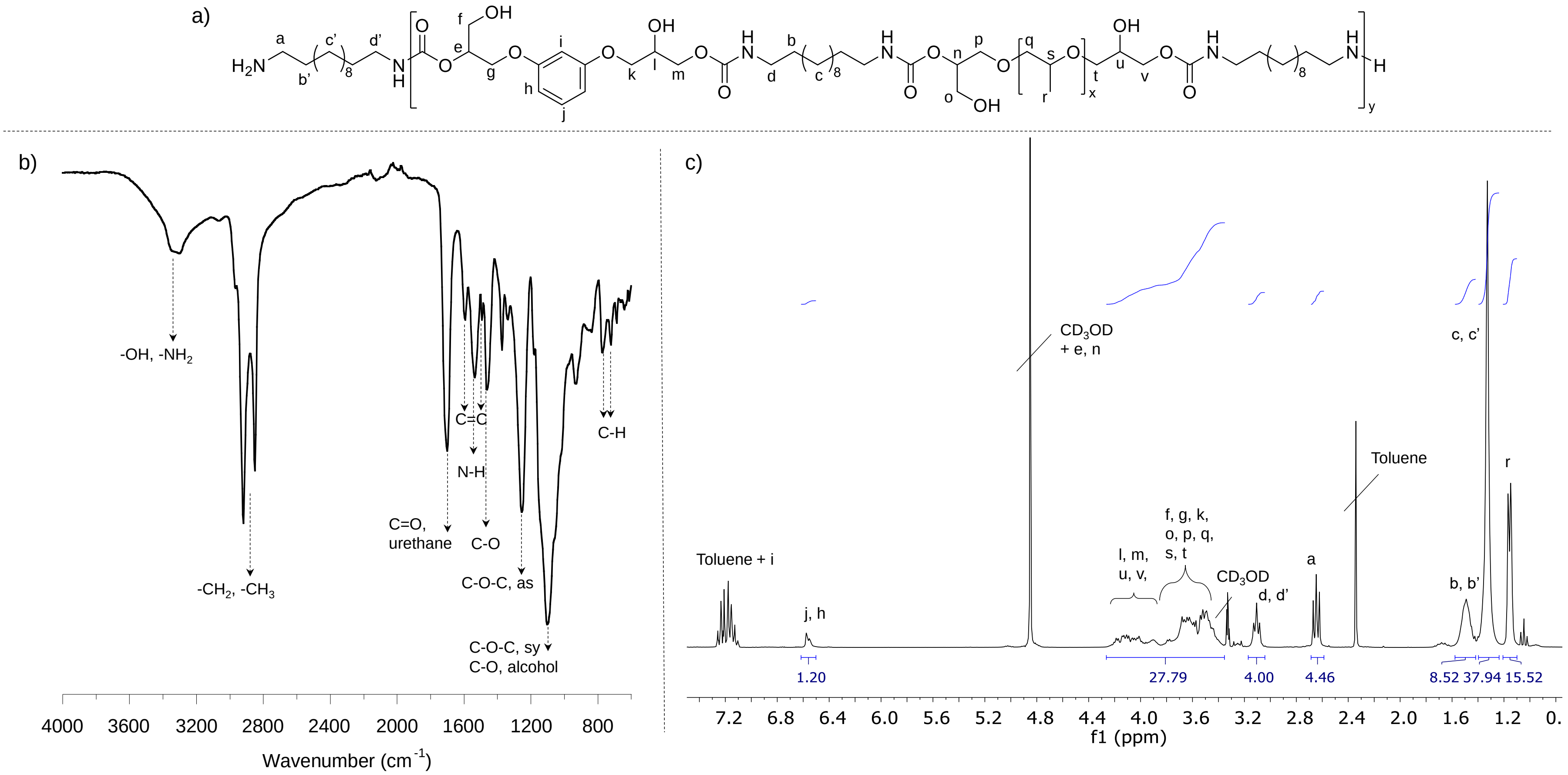

Figure S1. a) NH_2-PHU-NH_2 polymer structure; b) FTIR-ATR and c) ^1H NMR spectrum of the NH_2-PHU-NH_2.

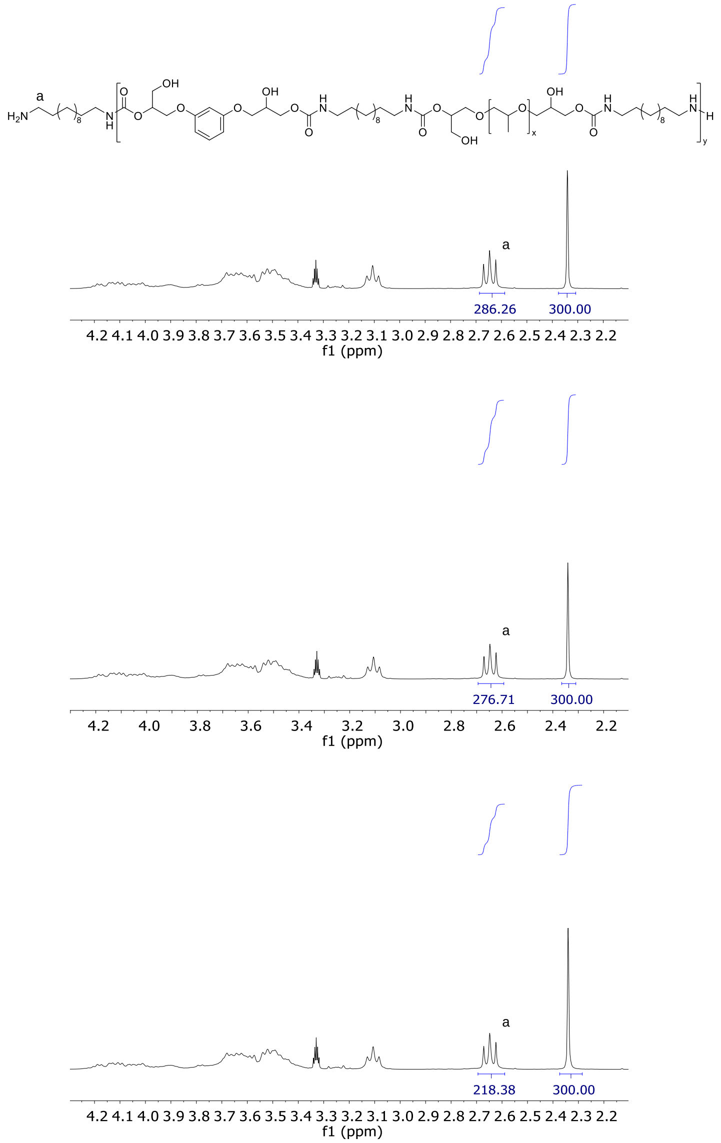

Figure S2. ^{1}H NMR spectra for triplicate of amine titration of NH_2-PHU-NH_2. The integration fixed to 300 correspond to the CH_3 signal of toluene added to the CH_3OH-d_4 standard solution. Rest of the signal of the spectra are assigned in Figure S1. The following masses were employed: spectrum 1 – $m_{NH2\text{-}PHU\text{-}NH2}$ = 56,8 mg, $n_{toluene}$ = 7.6 10^{-2} mmol; spectrum 2 – $m_{NH2\text{-}PHU\text{-}NH2}$ = 55,3 mg, $n_{toluene}$ = 7.8 10^{-2} mmol; spectrum 3 – $m_{NH2\text{-}PHU\text{-}NH2}$ = 51,1 mg, $n_{toluene}$ = 9.0 10^{-2} mmol.

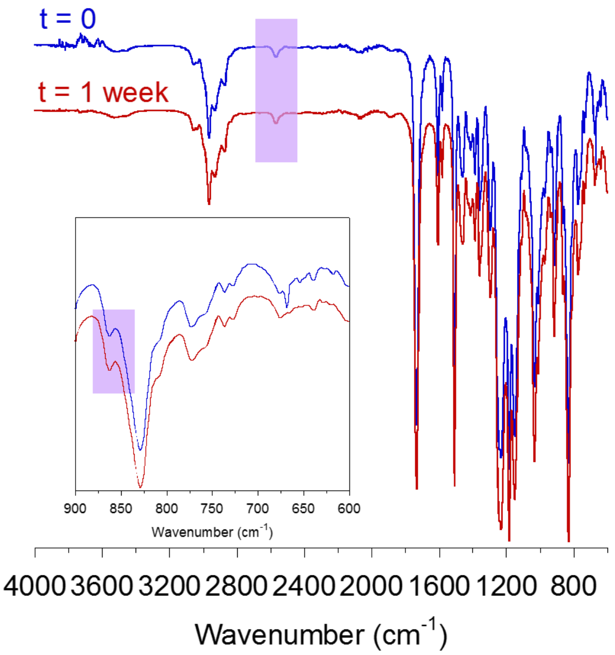

Figure S3. FTIR-ATR spectra of the mixture TMPTMP and EPIKOTE™ 828 at t = 0 and after one week kept at room temperature. Remaining vibration bands at 2569 cm^{-1} (S-H) and at 862 cm^{-1} (as C-O-C, epoxy), highlighted with purple, showed the stability of the mixture. In the box is zoomed between 900 and 600 cm^{-1} of the spectra.

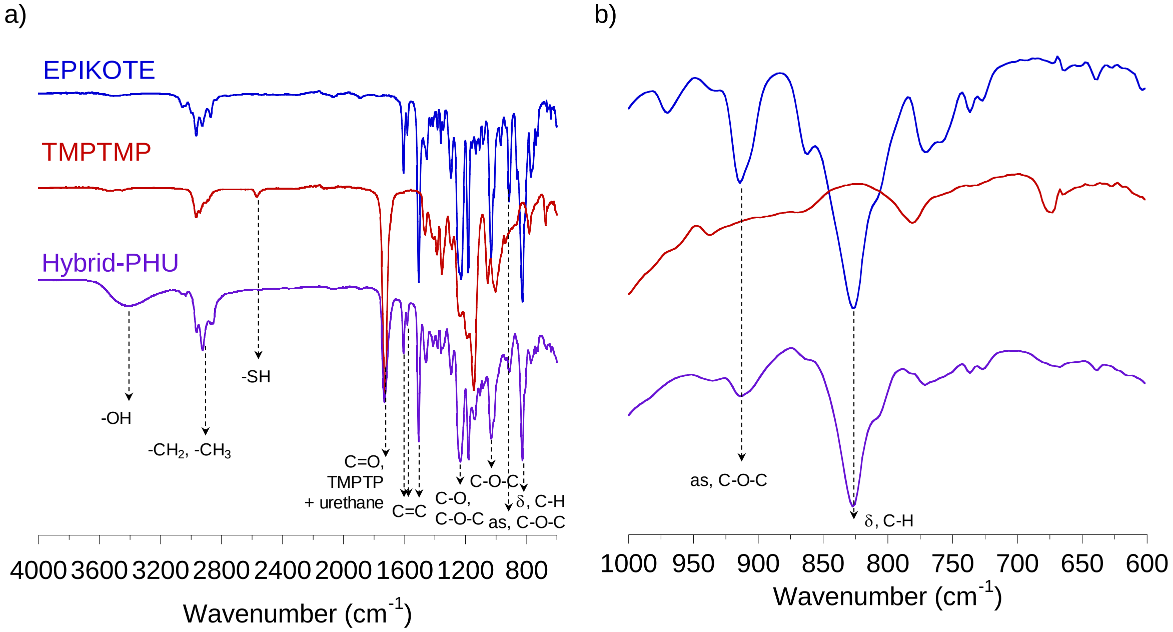

Figure S4. FTIR-ATR spectra of the curing process for 30/70 NH_2-PHU-NH_2/TMPTMP as representative example of the evolution of the curing process of hybrid PHU-epoxy adhesives.

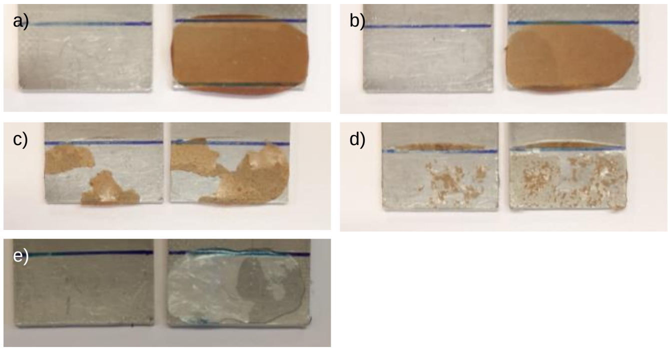

Figure S5. Photos of the representative failure nature of the PHU-epoxy hybrid compositions cured at room temperature for 24 h. a) 100/0, b) 70/30, c) 50/50, d) 30/70 and e) 0/100 NH_2-PHU-NH_2/TMPTMP equivalent ratios.
